# Supplementary material for: Intelligent in-cell electrophysiology: Reconstructing intracellular action potentials using a physics-informed deep learning model trained on nanoelectrode array recordings
Source: Nat Commun. 2025 Jan 14;16:657. doi: 10.1038/s41467-024-55571-6 (PMC11733287; doi:10.1038/s41467-024-55571-6)
Supplement: Supplementary file 1 — Supplementary Information [file 41467_2024_55571_MOESM1_ESM.pdf]

Supplementary Information (SI)

Supplementary Note 1*: Assessing the accuracy of NEA recordings when compared to patch clamp*

Ensuring the accuracy of the training data is vital for the effectiveness of any deep learning model. To determine the accuracy of iAP waveforms obtained from NEAs, we performed simultaneous NEA and patch clamp recording from the same cell and compared various features of their waveforms. Nano Crown-shaped NEAs (referred to simply as NEAs herein) were fabricated as previously reported [1]. iPSC-CM cells were then seeded onto the NEAs employing a differentiation and culture protocol as outlined in the Methods section.

We performed 15 sets of simultaneous iAP recordings from single cells, utilizing both patch clamp and NEA methods, with a comparative analysis of their waveforms shown in Figure-S1-a. Each recording set, as depicted in Figure-S1-b, demonstrated that although NEA iAP traces exhibit a lower amplitude, they closely align with the patch-clamp recordings when normalized. This normalization process allowed for a more accurate comparison between the two techniques. The total duration of these simultaneous recordings was approximately 46 minutes. To quantify the similarity between waveforms, we initially applied high-pass and low-pass filtering, as detailed in the Methods section, to decompose the waveform into isolated noise and refined action potential waveform. To account for various iAP shapes collected during recording, the signals were then segregated using a window length of 8000 timepoints or 1.6 seconds.

This process yielded a total of 3363 pairs of iAP recordings from both NEA and patch clamp methods. The S/N for both NEA and patch recordings was calculated by utilizing the filtered signal and noise vectors, assigning an S/N value to each window of the respective recording methods. Each window, containing pairs of action potential signals, was then normalized to a scale ranging from 0 to 1 to facilitate a more precise comparison. Furthermore, we quantified the APD values and their differences within each window, along with the cycle time for each recording (Figure-S1-c). We also calculated the discrepancy in cycle times between the NEA and patch clamp methods. Additionally, we determined the Mean Absolute Error (MAE) and analyzed the correlation (r) within each window (Figure-S1-d). This comprehensive analysis, which included comparisons of NEA and patch clamp recordings as depicted in the Figure-S1, provided an in-depth evaluation of the compatibility between these two recording techniques.

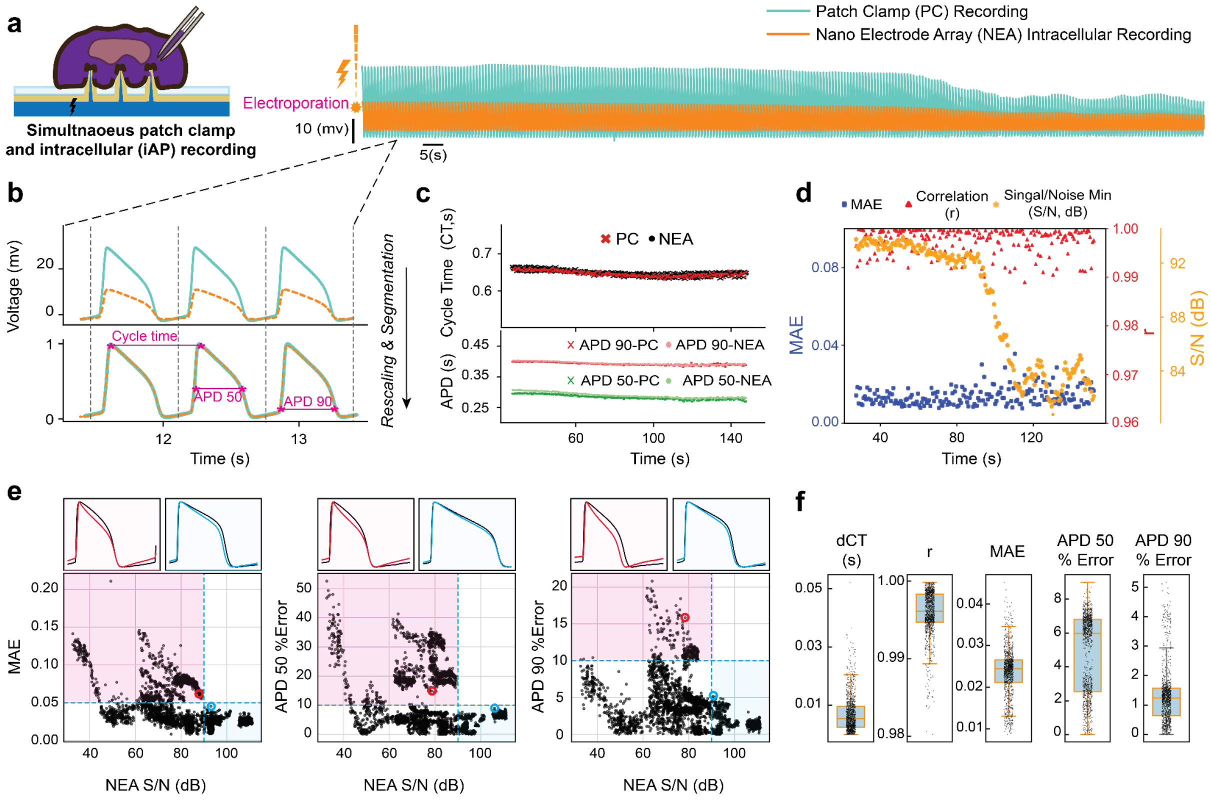

Figure-S1. a) Simultaneous iAP recording from iPSC-CMs using patch clamp (PC) and nano electrode array (NEA) via electroporation. **b)** Comparison of iAP recordings from PC and NEA: Scaling between 0 to 1 and segmenting into arrays of length 8000 indices or 1.6s. Includes important features such as cycle time, APD50 (action potential duration at 50% repolarization), and APD90. **c)** Comparison between windows of iAP from PC and NEA by cycle time, APD50, and APD90. **d)** Illustration of mean absolute error (MAE), correlation coefficient (r), and NEA signal to noise ratio (S/N) changes over time during one set of the experiment. **e)** Comparison between MAE, APD50% error, and APD90% error vs NEA S/N. It highlights the changes in three critical errors describing the similarity of NEA-recorded and PC-recorded normalized iAPs (MAE, APD50 percentage error, and APD90 percentage error), with thresholds set at 0.05, 10%, and 10% respectively to ensure reasonable similarity between iAP pairs. Also included is the comparison between iAP pairs with the highest MAE with NEA S/N > S/N threshold (S/N*), shown in blue, as well as examples of iAP pairs with error exceeding the threshold from the region with NEA S/N < 90. **f)** Box plot distribution of cycle time difference (dCT(s), r, MAE, APD50%, and APD90% errors between PC and NEA normalized iAP pairs with NEA S/N > S/N* (= 90) (n = 996). The box plots show the median (center line), interquartile range (IQR; box bounds), whiskers (1.5×IQR), and outliers (points beyond whiskers). The study conducted on n=3363 samples from 15 unique experiments before applying the S/N threshold.

The measured low MAE of 0.046 ±0.028 and a high average correlation (r) of 0.989 ± 0.012 pointed to the near-perfect agreement between the NEA iAP and patch-clamp recordings. Further, key parameters linked to drug-induced heart rhythm abnormalities were investigated by assessing iAP measurements from NEAs against patch clamping, focusing on APD50, and APD90 (cell repolarization markers) and cycle time . The average errors for APD50, and APD90 between NEA iAPs and patch clamps during the experiment were 0.032±0.034(s), and 0.011±0.016(s), respectively. Expressed as percentage errors, these values equate to 11.516±10.010%, and 3.875±3.425%, respectively. For cycle time, the mean difference between two consecutive spikes was 0.011 ± 0.024 (s). Figure-S1-e illustrates how the MAE, APD 50, and APD 90 errors vary across a range of values depending on the S/N in NEA recording traces.
The figure highlights that as the NEA S/N ratio increases, the maximum values of these errors decrease. This trend suggests that low S/N ratios may cause distortions in iAP shape, potentially due to an imperfect cell-to-NEA seal[1]. These distortions seem to follow a probability distribution dependent on the S/N value, with deviations diminishing at higher S/N ratios. Based on these findings, to ensure waveform accuracy,

we set maximum acceptable thresholds at 0.05 for MAE, and 10% for both APD 50 and APD 90 percentage errors. Our analysis revealed that applying a stringent threshold of 90 dB (S/N* =90 dB) and filtering signals with S/N ratios above S/N* ensured that the APD 50 percentage error and MAE remained below 10% and 0.1, respectively, as demonstrated in Figure-S1-e. The figure also presents examples of eAP and iAP pairs that illustrate the maximum errors observed at or above the S/N*.

Upon comparing NEA iAPs that satisfied the S/N* with corresponding patch clamp iAP recordings, we observed a significant reduction in errors. MAE decreased to 0.024±0.006, and the average correlation coefficient (r) increased to 0.996 ± 0.003. The average errors for APD50 and APD90 between NEA iAPs and patch clamps improved to 0.017±0.020 seconds and 0.004±0.007 seconds, respectively. When expressed as percentage errors, these values correspond to 4.815±2.342% for APD50 and 1.343±0.993% for APD90. Additionally, the mean cycle time difference between two consecutive spikes was recorded at 0.007 ± 0.006 seconds. The distributions of aforementioned errors are shown in Figure-S1-f. Furthermore, the experiment level comparison of normalized iAPs from NEA recording and patch clamp is provided in Figure-S2. These findings align with a comprehensive comparison of NEA and patch clamp iAPs, particularly when various drugs were introduced during recordings [1]. Our findings revealed a near-perfect match between iAPs from the two recording methods when the S/N* was exceeded. We used this S/N* threshold for processing of data used to train our model in subsequent steps.

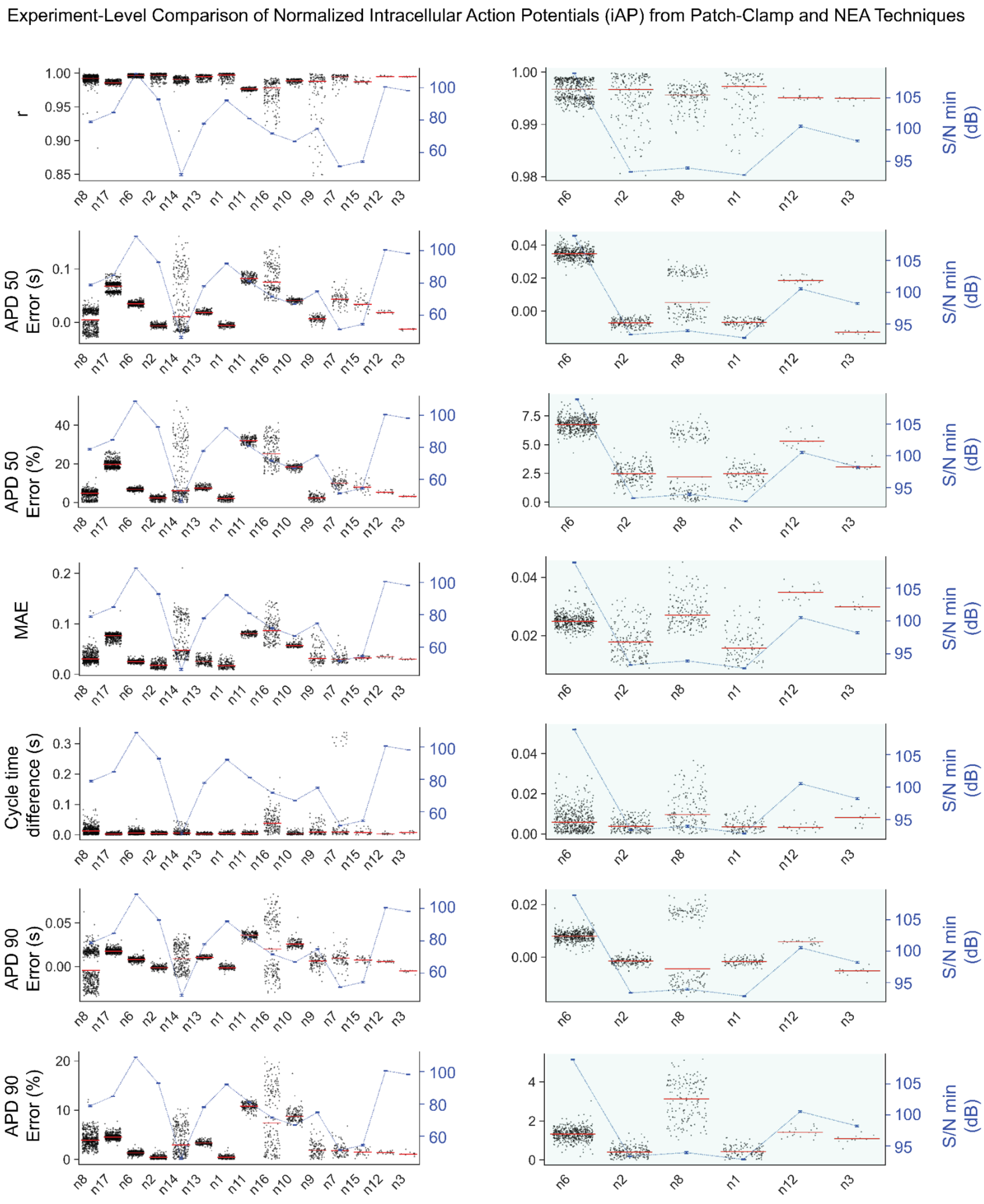

Figure-S2. Experiment-level comparison of normalized iAP from patch clamp (PC) and nano-electrode array (NEA) recording techniques. Fifteen unique sets of successfully simultaneous iAP recordings using NEA and PC are presented, showcasing various errors (r, APD 50, APD 90, MAE, cycle time difference, APD 50%, and APD 90%). The left panel displays comparisons for all recordings, while the right panel focuses on recordings with NEA iAP signal-to-noise ratio (S/N) greater than 90 and comprising more than 10 samples. The red lines show the median value. The study conducted on n=3363 samples from 15 unique experiments before applying the S/N threshold.

Supplementary Note II: *Two neighboring channels on NEA show similar iAPs.*

To obtain synchronized eAP and iAP recordings from NEAs, we assumed that neighboring cells in a confluent monolayer of iPSC-CMs that are in close physical proximity on the NEAs exhibit similar AP waveform. We tested this assumption by thoroughly assessing the similarity between neighboring channels over an extended period of time, and under various conditions (Figure-S3). To this end, we incrementally introduced Dofetilide (dissolved in DMSO) – a compound known to prolong iAP duration [2] – into the cell culture while recording iAP signals from many channels. The drug's concentration was progressively increased from 0.3 to 1, then to 3, and finally to 10 nM through a multi step addition process. This process was done in four stages, occurring approximately at 400, 800, 1200, and 1700 seconds during the recording session, as illustrated in Figure-S3-a. Dofetilide addition enables us to study the similarity between two neighboring channels for a wider range of iAP durations. In total, 2661 pairs for iAP spikes from 22 pairs of simultaneous recordings from neighboring channels were collected with S/N > 90 (dB). We applied the same similarity analysis we conducted for NEA versus patch clamp iAPs. We scaled paired NEA neighboring channels iAPs to a range of 0 to 1 (Figure-S3-b), then calculated the MAE, and correlation between them along with comparing their APD 50, APD 90 and cycle time (Figure-S3-c). The average difference (MAE) across two iAP traces was 0.011 ± 0.006. Moreover, the correlation coefficients (r) between the iAPs two neighboring channels were quantified as 0.999 ± 0.001 respectively. The average errors for APD50, and APD90 between two neighboring NEA channels iAPs were 0.015 ± 0.009(s), and 0.006 ± 0.005(s), respectively. Expressed as percentage errors, these values equate to 3.943 ± 2.233%, and 3.943 ± 2.233 %, respectively. For cycle time, the mean difference between two consecutive spikes was 0.002 ± 0.002 (s).

Moreover, Figure-S3-d exhibits pairs of iAPs from neighboring channels, highlighting the maximum MAE, APD 50 percentage error, and APD 90 percentage error observed. This evidence underscores the consistency in iAP traces from neighboring NEA channels when the S/N ratio is greater than the S/N*. This noteworthy outcome underscores the potential to use two neighboring channels to record synchronously from two adjacent cells in a confluent monolayer effectively obtaining both eAP from one channel and iAP from the other, as if acquiring both from a single cell. Leveraging this understanding, in the next step, we electroporated a cell through one channel while recording eAP from the adjacent cell, enabling the simultaneous capture of both eAP and iAP data. This approach was implemented to record the input (eAP) and corresponding output (iAP) data of our data analysis, machine learning, and deep learning models.

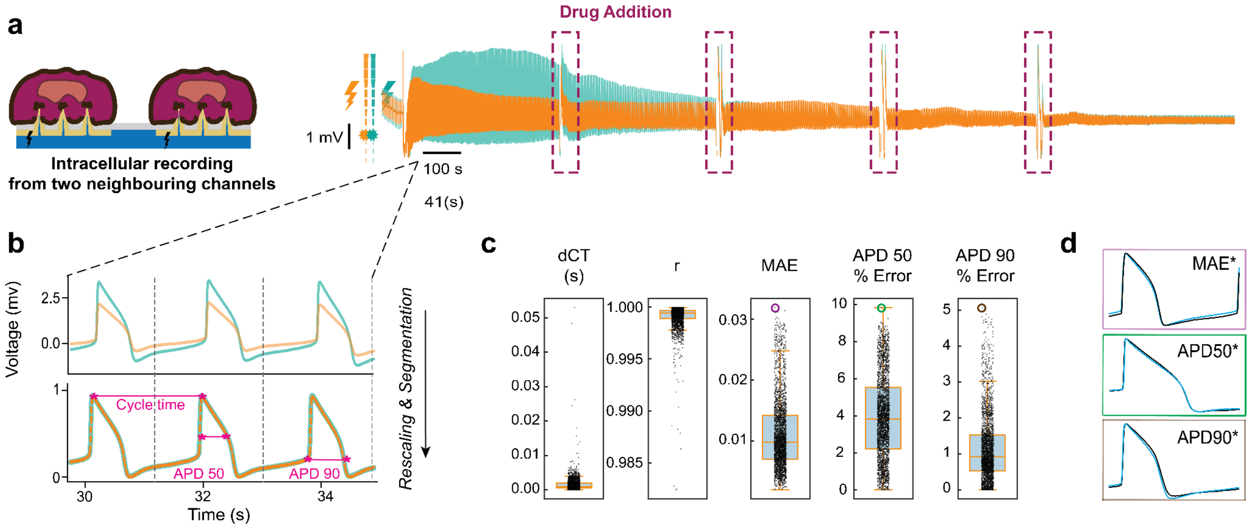

Figure-S3. a) Simultaneous iAP recording from neighboring channels via non-invasive extracellular action potential (NEA) electroporation. This section details the multi-step addition process of Dofetilide, administered in concentrations of 0.3, 1, 3, and finally 10 nM. The process was conducted in four stages at approximately 400, 800, 1200, and 1700 seconds during the recording session. This method was employed to collect a diverse range of iAP shapes, facilitating a more comprehensive analysis. **b)** Comparison process similar to PC vs NEA iAP recording: Scaling between 0 to 1 and segmenting into arrays of length 8000 indices or 1.6s. **c)** Box plot distribution of dCT(s), correlation coefficient (r), MEA, and APD50% and APD90% errors between neighboring and NEA normalized iAP pairs with NEA S/N > S/N* (= 90) for n = 2661 samples, comparing 22 pairs of neighboring iAP channels from two independent cell cultures. The box plots show the median (center line), interquartile range (IQR; box bounds), whiskers (1.5×IQR), and outliers (points beyond whiskers). **d)** Examples of iAP pairs from neighboring channels with the highest MEA, APD50, and APD90% errors, as indicated in the box plots.

The experiment-level comparison of normalized iAPs from neighboring NEA channels is shown in Figure-S4.

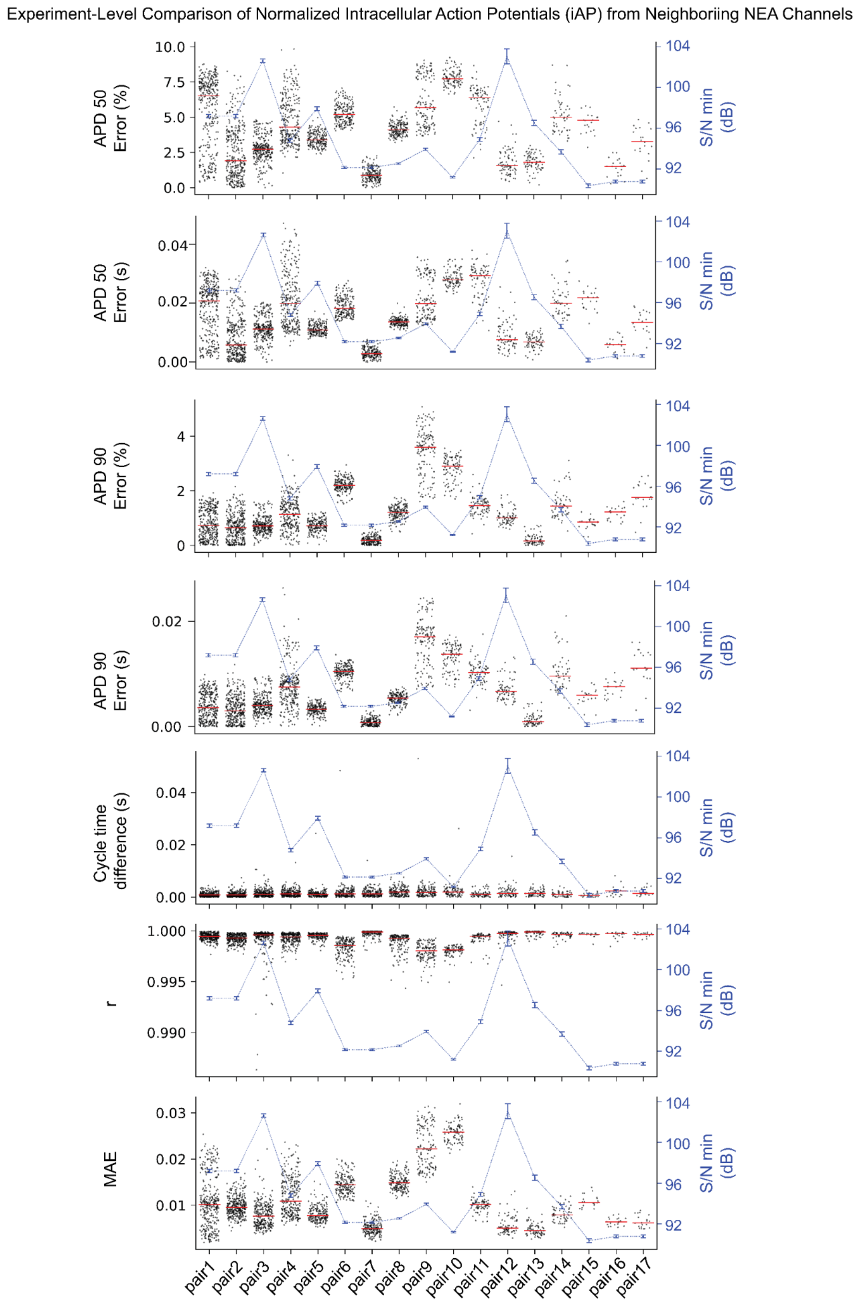

Figure-S4. Experiment-level comparison of normalized iAPs from neighboring NEA channels with a minimum S/N > 90, involving 17 pairs of channels recorded in two distinct experiments. This comparison highlights various errors, including correlation coefficient (r), APD 50, APD 90, mean absolute error (MAE), cycle time difference, and percentage errors for APD 50% and APD 90%. for n = 2661 samples, comparing 22 pairs of neighboring iAP channels from two independent cell cultures. The red lines show the median value.

It should be noted that recording from the same electrode during repetitive stimulation to obtain two consecutive identical iAPs, rather than using neighboring channels for iAPS, presents two main challenges with this approach:

A. **Gradual Change in iAP Shapes**: When cells are exposed to drugs, the iAP shapes can gradually change over time. As a result, two subsequent iAPs may not necessarily be similar, especially if the drug effects are still evolving. This makes it difficult to ensure that the recorded iAPs are truly identical or representative of a steady-state condition.
B. **Electroporation Recovery Time**: After a cell is electroporated, it typically takes around 30 to 60 minutes for the membrane to reseal and recover fully. If we were to record an eAP) followed by the next iAP after electroporation, the 30-minute waiting period would significantly reduce the throughput of data recording. This slow process would hinder our ability to collect the large number of synchronized eAP/iAP pairs necessary for training deep learning models, which require substantial amounts of data.

Supplementary Note III: *Drug test experiments.*

The following provides a detailed overview of the drugs used in the experiments, their interactions with cardiac ion channels, and their impact on the shape of action potentials.

Dofetilide: A Class III antiarrhythmic drug, dofetilide primarily blocks the hERG (IKr)[37] potassium channels. By inhibiting this channel, it prolongs the repolarization phase, resulting in an increased action potential duration (APD) and a longer QT interval (Jaiswal and Goldbarg 2014).

Quinidine: As a Class IA antiarrhythmic, quinidine blocks sodium (Na^+) channels (INa), effectively depressing the rapid initial depolarization phase of the action potential. This mechanism reduces the sodium current and affects the overall electrical activity of the heart (Imaizumi and Giles 1987)

Nifedipine: A calcium channel blocker in the dihydropyridine class, nifedipine selectively blocks L-type calcium channels (ICa-L)[41]. This results in a shortened plateau phase of the action potential, leading to a reduced action potential duration (Zemzemi and Rodriguez 2015)

Flecainide: A Class IC antiarrhythmic drug, flecainide blocks both Na^+ channels (INa) and, to a lesser extent, potassium (IKr) channels [42,43] . Its overall effect is to prolong the duration of the action potential and the effective refractory period in ventricular fibers, while both are shortened in the Purkinje system, which is likely consistent with its sodium channel blockade (Lavalle et al. 2021)

Lidocaine: A Class IB antiarrhythmic, lidocaine primarily blocks Na^+ channels (INa)[44] . It may shorten the APD, though the effect observed in the experiments varied based on the sample conditions.

Propranolol: Known as a beta-blocker [45] and classified as a Class II antiarrhythmic, propranolol also exhibits sodium (INa) channel blocking properties, further influencing cardiac electrical activity (Kirkorian et al. 1988). It can result in shortening APD 90, by increasing the repolarization slope(Hirose et al. 2020).

This comprehensive understanding of the drug-channel interactions and their resultant impact on action potentials aids in evaluating the electrophysiological responses during experiments.

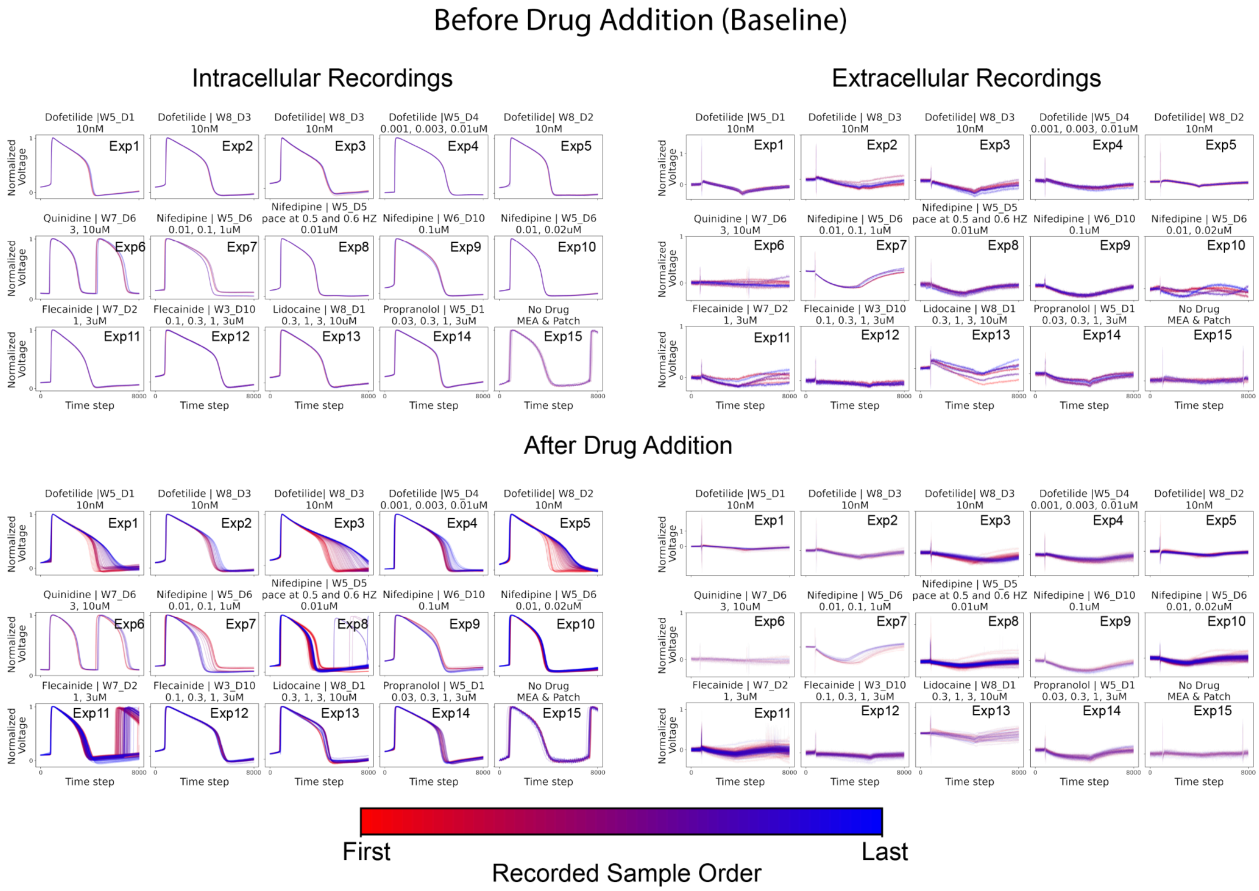

Figure-S5. Comparison of eAP and iAP baseline recordings across different experiments, as well as a comparison of the drugs used in the study, their dosages, and their impact on normalized iAP shapes. Additionally, we include the wafer (W) and device (D) numbers of the unique Nano Electrode Arrays used in the study. A color gradient from red to blue indicates the timeline of the experiments, with the bluer tones representing data from more recent experiments.

Supplementary Note IV: *eAP Features Determination Methods.*

Determination of Starting Point bp_1: bp_1, is identified through a series of signal processing steps aimed at detecting significant changes in the signal's behavior.

1. **Signal Smoothing:** The initial portion of the extracellular action potential (eAP) signal, from the beginning up to the point of maximum voltage (x1), is extracted for analysis. To reduce noise and highlight the essential features of the signal, a Savitzky-Golay filter is applied. This filtering technique smooths the signal while preserving its overall shape, which is critical for accurately detecting transitions in the signal.
2. **Gradient Calculation:** After smoothing, the gradient of the signal is computed to accentuate the areas where rapid changes occur, which are often indicative of important transitions such as the

start of the signal. This gradient is further smoothed to reduce noise, which ensures that only the most significant changes are considered in the next step.

3. **Change Point Detection:** The processed gradient signal is analyzed using a method based on Jenks natural breaks optimization, which is designed to find natural divisions in data by minimizing the variance within groups and maximizing the variance between groups. In this context, the method is used to identify distinct changes in the signal that likely correspond to the initiation of the eAP. Specifically, the algorithm segments the gradient into three distinct classes, and the most relevant change point for bp1 is selected as the last point where the gradient signal shifts from one class to another.

Determination of bp_2: bp_2, is determined using a similar methodology, tailored to identify the conclusion of the signal.

1. **Signal Segmentation:** A specific segment of the eAP signal, starting just beyond the point of maximum voltage and extending a short distance thereafter, is isolated for this analysis. This region is chosen because it is expected to contain the signal's descent back to the baseline, marking the end of the action potential.
2. **Signal Smoothing and Gradient Calculation:** As with the determination of bp1, the signal segment is smoothed using the Savitzky-Golay filter, and its gradient is calculated and smoothed. This step is crucial for emphasizing the downward trend as the signal returns to baseline levels.
3. **Change Point Detection:** The processed gradient of this segment is then analyzed using a slightly modified version of the Jenks natural breaks optimization, this time dividing the data into two classes. This adjustment reflects the simpler nature of the signal at this stage, which primarily involves a transition back to the baseline. The algorithm identifies the most significant change point as the first point where the gradient signal transitions between classes, corresponding to the point where the signal's descent begins to level off, marking the end of the eAP.

Supplementary Note V: *eAP features screening.*

Distorted spikes in eAP signals, as shown in Figure 2b, and the strong correlation between ΔT_1 and ΔT_2 with ΔT_s (r = 0.93 and r = 0.87 respectively), led us to opt ΔT_s the representative of the eap spike temporal features.

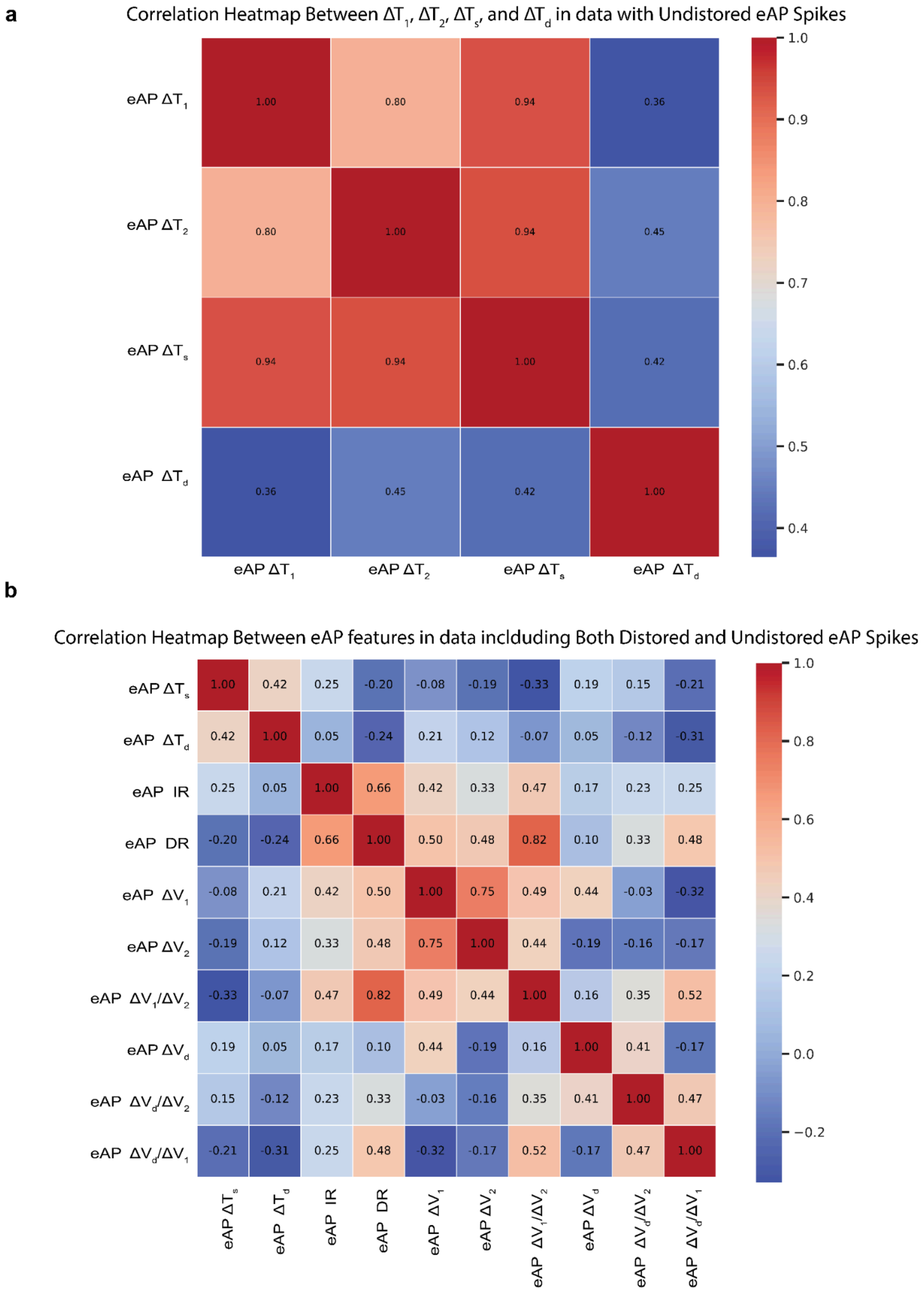

Figure-S6. a) Correlation between temporal-related eAP features (ΔT_1, ΔT_2, ΔT_s, and ΔT_d) for the undistorted portion of eAP/iAP data collected to be utilized in the machine learning and deep learning models. **b)** Correlation between measurable eAP features (excluding ΔT_1 and ΔT_2) for all of the eAP/iAP data (with distorted and undistorted eap spikes) utilized in the machine learning and deep learning models (n = 1049) samples..

Supplementary Note VI:*Physics loss in PIA-UNET.*

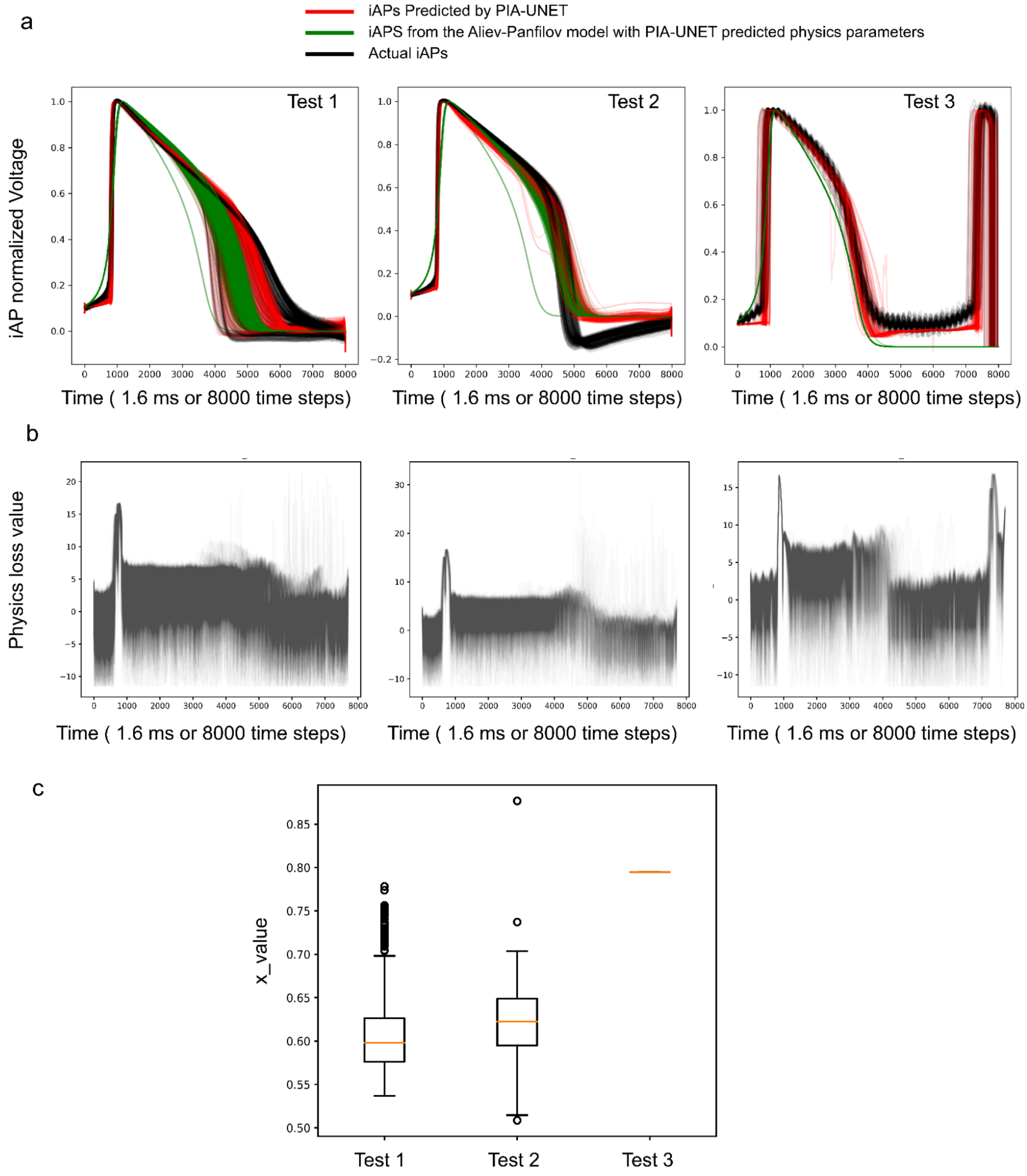

Figure-S7. **a)** Overlay of predicted iAPs using PIA-UNET, actual iAPs, and iAPs generated by the Aliev-Panfilov model based on PIA-UNET physics parameter output values, for all three test sets. **b)** Overlay of the physics-informed loss values from the PIA-UNET for all three test set predictions. The parameter ***a*** was predicted to be 0.057, ***k*** was fixed at 100.00 for all samples, and the distribution of ***x*** is shown in part c. The initial membrane potential u_0 was set to 0.1, with ***du/dt*** at t_0 initialized to 0.4 and the

equation was solved for t = 1.6 sec. The box plots show the median (center line), interquartile range (IQR; box bounds), whiskers (1.5×IQR), and outliers (points beyond whiskers).

Supplementary Note VII: *Table S1 – Comparison of Model Performance With and Without Physics-based Loss Incorporation*

Table S1

**PHYSICS**	***MEA Test1***	***MEA Test2***	***MEA Test3***	***M-APD Test1 [ms]***	***M-APD Test2 [ms]***	***M-APD Test3 [ms]***	***M-APD Test1 [%]***	***M-APD Test2 [%]***	***M-APD Test3 [%]***	***Runs***
**ON**	0.036 ± 0.004	0.051 ± 0.004	0.040 ± 0.002	0.030 ± 0.001	0.022 ± 0.002	0.023 ± 0.006	5.782 ± 0.582	4.216 ± 0.315	4.915 ± 1.199	***3***
**OFF**	0.035 ± 0.001	0.029 ± 0.003	0.048 ± 0.002	0.033 ± 0.002	0.023 ± 0.002	0.033 ± 0.006	7.902 ± 0.394	4.772 ± 0.360	6.579 ± 1.090	***3***

The results, reported as mean ± standard deviation

The results show that incorporating physics into the loss function led to a notable improvement in APD error, enhancing the waveform similarity. However, this adjustment caused a higher mean absolute error due to the correction of super-repolarization—a brief undershoot at the end of repolarization observed in some NEA recordings. In terms of generalizability, the physics-based approach performed significantly better in Test 3, which involved eAPS on MEA, when compared to patch clamp iAPS recordings. This suggests improved robustness across different test conditions despite the increase in absolute error.

Section VIII: *NanoElectrode Arrays Layout and Neighboring Channels Distance*

The following figure shows the typical distance between two neighboring channels that we used to record pairs of iAP/eAP signals. The average distance between these channels is approximately 120 micrometers.

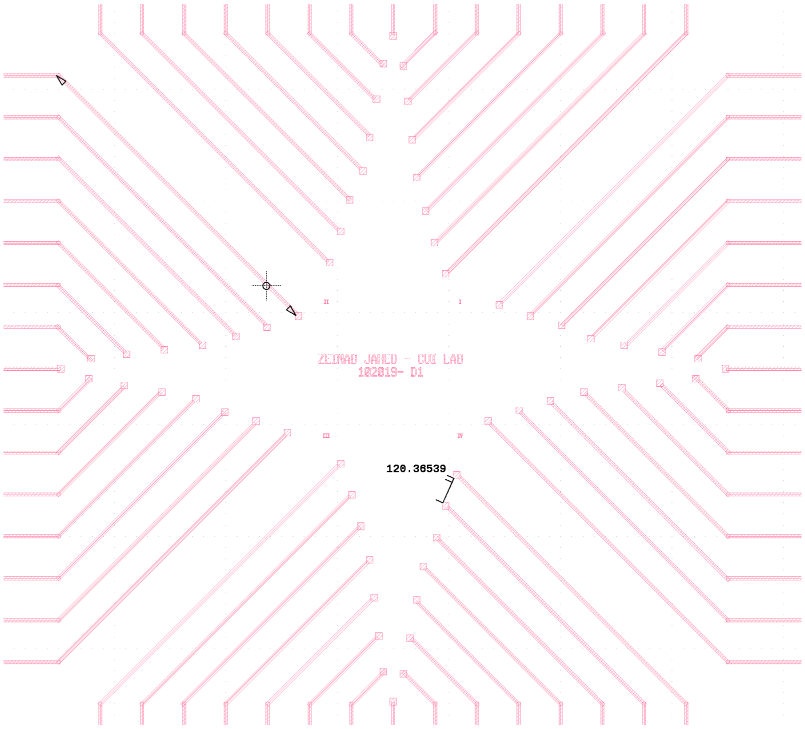

Figure-S8. NEA layout showing an example of the distance between two neighboring channels.

Supplementary Note IX*: Activation Map and APD Map*

To study the application of PIA-UNET on NEA multi-channel recordings, the APD map and Activation map are calculated. However, it should be noted that the channels are arranged in a star-shaped nanoelectrode dish, which must be considered when comparing the Activation Map and APD Map.

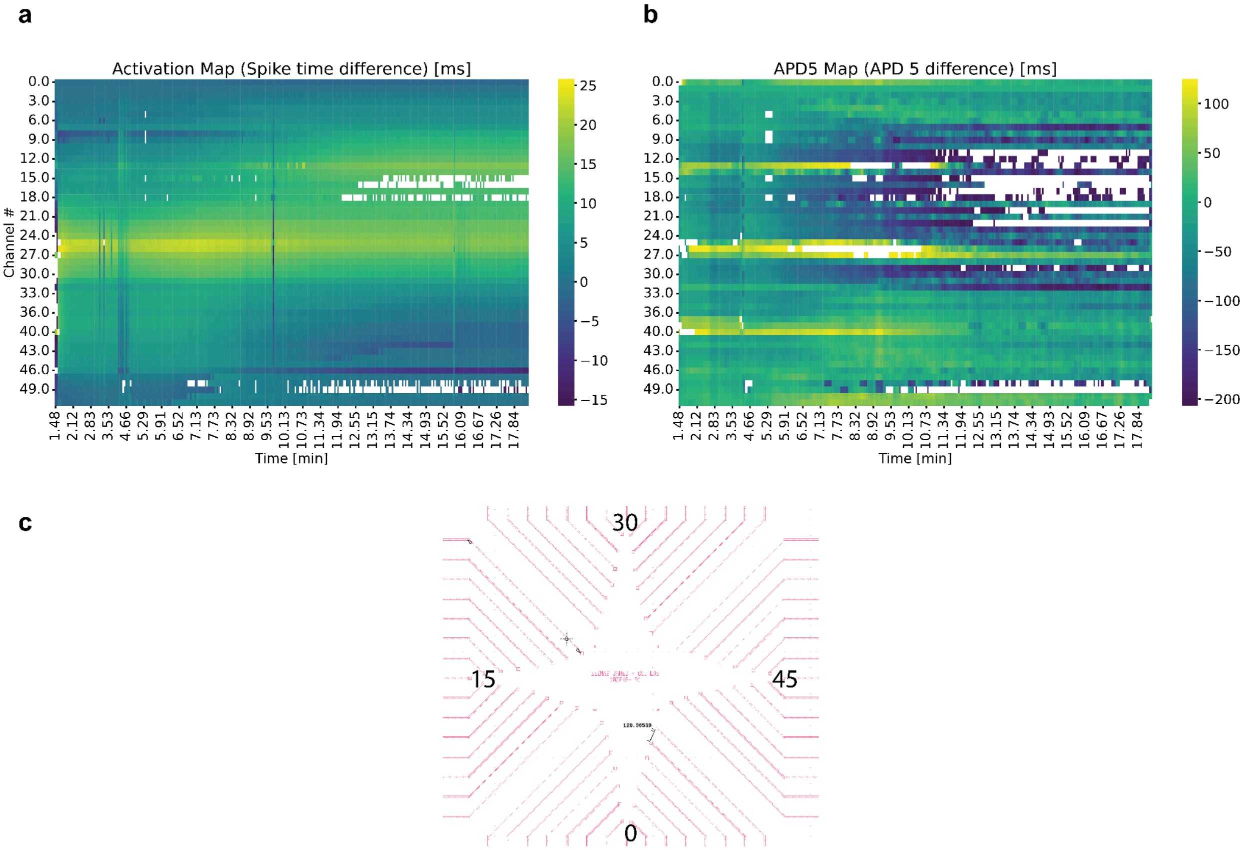

Figure-S9. a) NEA-recorded eAP Activation Map vs. **b)** its reconstructed APD 50 Map. Note that the channels are arranged in a star-shaped pattern, as illustrated in part **c** of the figure.

Supplementary Note X*: Simplification of the Aliev-Panfili Model*

We start with the following governing equations:

$$\frac{dv}{dt} = kv(1 - v)(v - a) - vw \ (1)$$

$$\frac{dw}{dt} = \varepsilon(v, a, x)(kv - w) \ (2)$$

with $\varepsilon(v, a, x)$ approximated by:

$$\varepsilon(v, a, x) \approx x.\sigma\,(n\,(a - v)) + (1 - x).\sigma\,(n\,(v - a)) \ (3)$$

1. Isolating w from Equation 2

We solve for w from Equation 12:

$$w = \frac{kv(1 - v)(v - a) - \frac{dv}{dt}}{v}$$

2. Substituting w into Equation 3

$$\frac{d}{dt}\left[\frac{kv(1 - v)(v - a) - \frac{dv}{dt}}{v}\right] = \varepsilon(v, a, x)\left(kv - \frac{kv(1 - v)(v - a) - \frac{dv}{dt}}{v}\right)$$

For $v \neq 0$, we divide by v and simplify.

$$\frac{d}{dt}[k(1 - v)(v - a) - v^{-1}\frac{dv}{dt}] = \varepsilon(v, a, x)(kv - [k(1 - v)(v - a) - v^{-1}\frac{dv}{dt}])$$

3. Simplification of the Left Side

$$\frac{d}{dt} = k\frac{d(v - a - v^2 + a.v)}{dt} - \frac{d[v^{-1}\frac{dv}{dt}]}{dt}$$

We differentiate each term:

$$k\frac{d}{dt}[(1 - v)(v - a)] = k(\frac{dv}{dt} - 2v\frac{dv}{dt} + a\frac{dv}{dt})(*)$$

and

$$-\frac{d[v^{-1}\frac{dv}{dt}]}{dt} = -\left(-\frac{dv}{dt}\frac{dv}{dt}v^{-2} + v^{-1}\frac{d^2v}{dt^2}\right)(**)$$

4. Putting These Together:

$$\frac{d}{dt}[k(1 - v)(v - a) - v^{-1}\frac{dv}{dt}] = k[\frac{dv}{dt} - 2v\frac{dv}{dt} + a\frac{dv}{dt}] + \left(\frac{dv}{dt}\right)^2 v^{-2} - v^{-1}\frac{d^2v}{dt^2}$$

This simplifies to:

$$= k\frac{dv}{dt}[1 - 2v + a] + \left(\frac{dv}{dt}\right)^2 v^{-2} - v^{-1}\frac{d^2v}{dt^2}$$

6. Putting Together the Left and Right Sides

$$F_{AP}(v, \frac{dv}{dt}, \frac{d^2v}{dt^2}, a, k) = v^{-2}(\frac{dv}{dt})^2 - v^{-1}\frac{d^2v}{dt^2} - \frac{dv}{dt}[\varepsilon(v)v^{-1} - k(1 - 2v + a)] - k\varepsilon(v)(v^2 - av + a) = 0$$

Supplementary Note XI: *Table S2 – XGBoost Model Performance on the Training and Test Sets*

Table S2

**Dataset**	**APD-30 (s)**	**APD-50 (s)**	**APD-70 (s)**	**APD-90 (s)**	**APD-30 (%)**	**APD-50 (%)**	**APD-70 (%)**	**APD-90 (%)**
**Train**	0.002 ± 0.002	0.002 ± 0.002	0.002 ± 0.002	0.002 ± 0.002	0.522 ± 0.570	0.385 ± 0.412	0.363 ± 0.386	0.344 ± 0.353
**Test 1**	0.018 ± 0.011	0.027 ± 0.013	0.023 ± 0.012	0.044 ± 0.020	4.526 ± 2.732	4.188 ± 1.961	2.838 ± 1.513	4.842 ± 2.165
**Test 2**	0.088 ± 0.026	0.056 ± 0.023	0.014 ± 0.009	0.028 ± 0.023	18.748 ± 5.309	8.295 ± 3.400	1.912 ± 1.184	3.585 ± 2.759
**Test 3**	0.020 ± 0.010	0.044 ± 0.037	0.066 ± 0.062	0.051 ± 0.066	5.096 ± 2.386	8.795 ± 7.411	11.763 ± 11.203	8.233 ± 10.746

The results, reported as mean ± standard deviation

Supplementary Note XII*: Violin plot distribution of eAP and iAP features for the data*

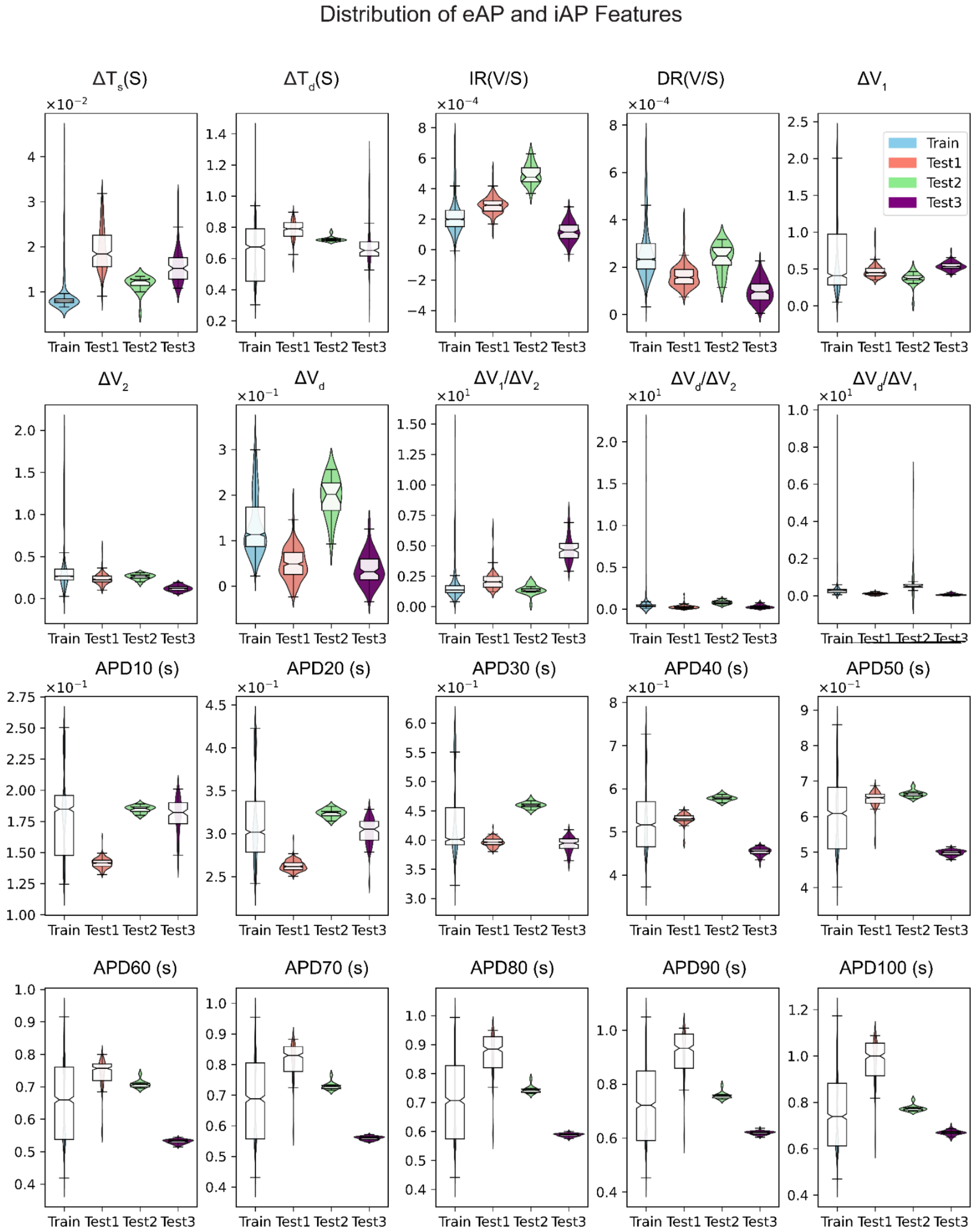

Figure-S10. Violin plot distribution of eAP and iAP features for the data utilized in the machine learning and deep learning models, as depicted in Figure-3b, shown in green. The blue and red plots represent the distribution of the training set and test set for eAP and iAP, respectively ($n_{training-val}$= 1209, n_{Test1}= 272, n_{Test2}= 171 and, n_{Test3}= 91).

Supplementary Note XIII: *Temporal Variations of eAP and iAP waverfroms*

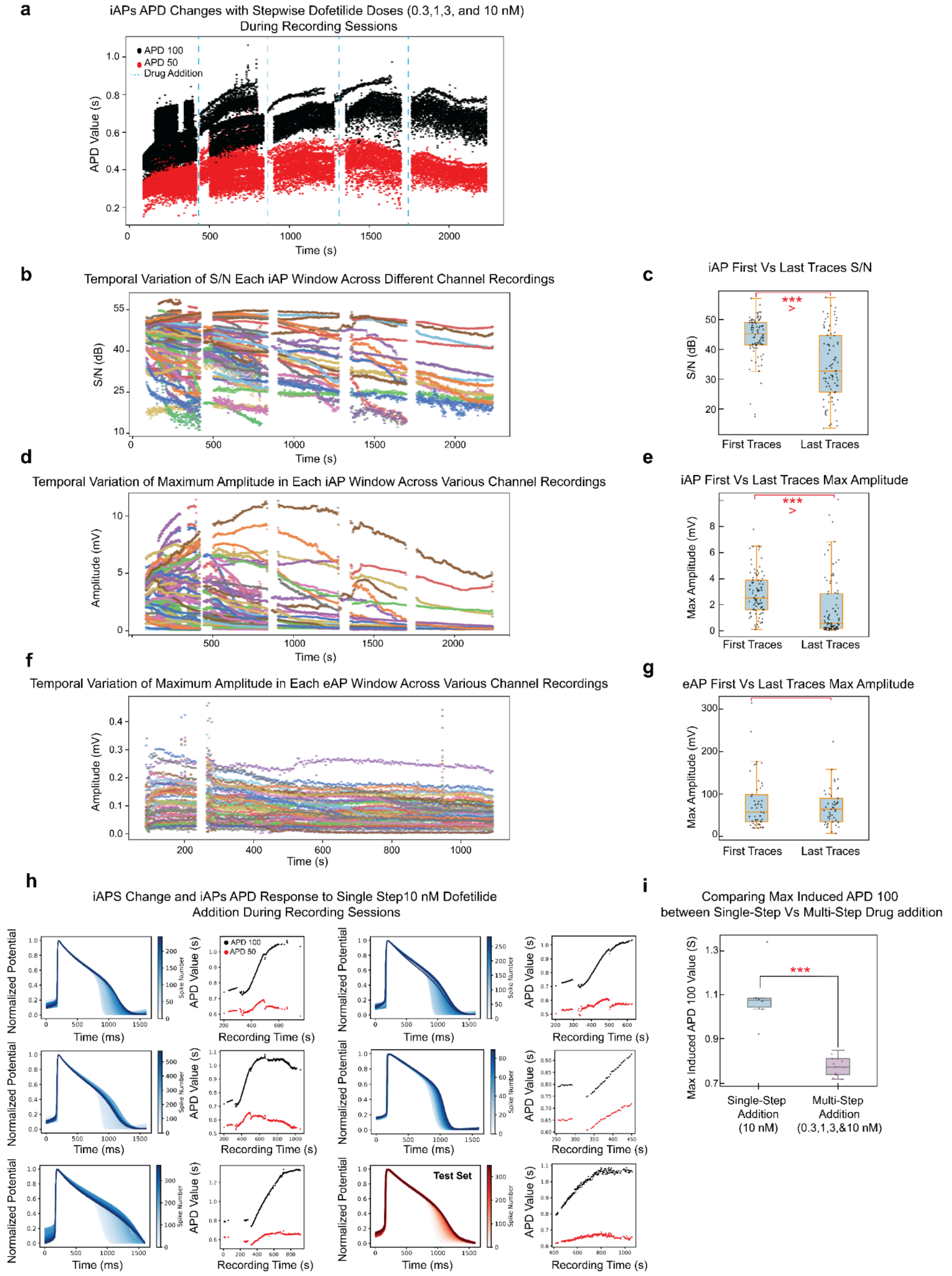

Figure-S11. **a)** Comparison of iAP APD values upon drug addition, based on experiments comparing neighboring NEA channels' iAPs, from 88 unique iAP channels across two sets of recordings. The dashed line indicates the time of drug addition. **b)** Showcase of the variation in iAP signal-to-noise ratio (S/N) over time from the same recordings as above. **c)** Comparison of the last iAP in the recording with the first one in terms of S/N, demonstrating a significant drop in value (P value = [***p-value: 8.63 x 10^{-15}]) as the experiment progresses, due to the resealing of cell membrane pores. **d)** Presentation of the variation in iAP window's maximum amplitudes (spike amplitude) over time from the same recordings as above. **e)** Comparison of the last iAP in the recording with the first one in terms of amplitude, showing a significant drop in value (***p- value = [1.1 x 10^{-08}]) as the experiment progresses, attributed to the resealing of cell membrane pores. **f)** Presentation of the variation in eAP maximum amplitude (spike amplitude) over time for 49 distinct eAP channels. **g)** Comparison of the last eAP in the recordings with the first one in terms of amplitude, indicating no significant change (p- value = [0.262]). **h)**iAPs collected from distinct sets of recordings by the addition of dofetilide, used for the machine learning and deep learning aspects of this study. The test set here corresponds to Test1.

Supplementary Note XIV: *Performance of Quantile Based PIA-UNET*

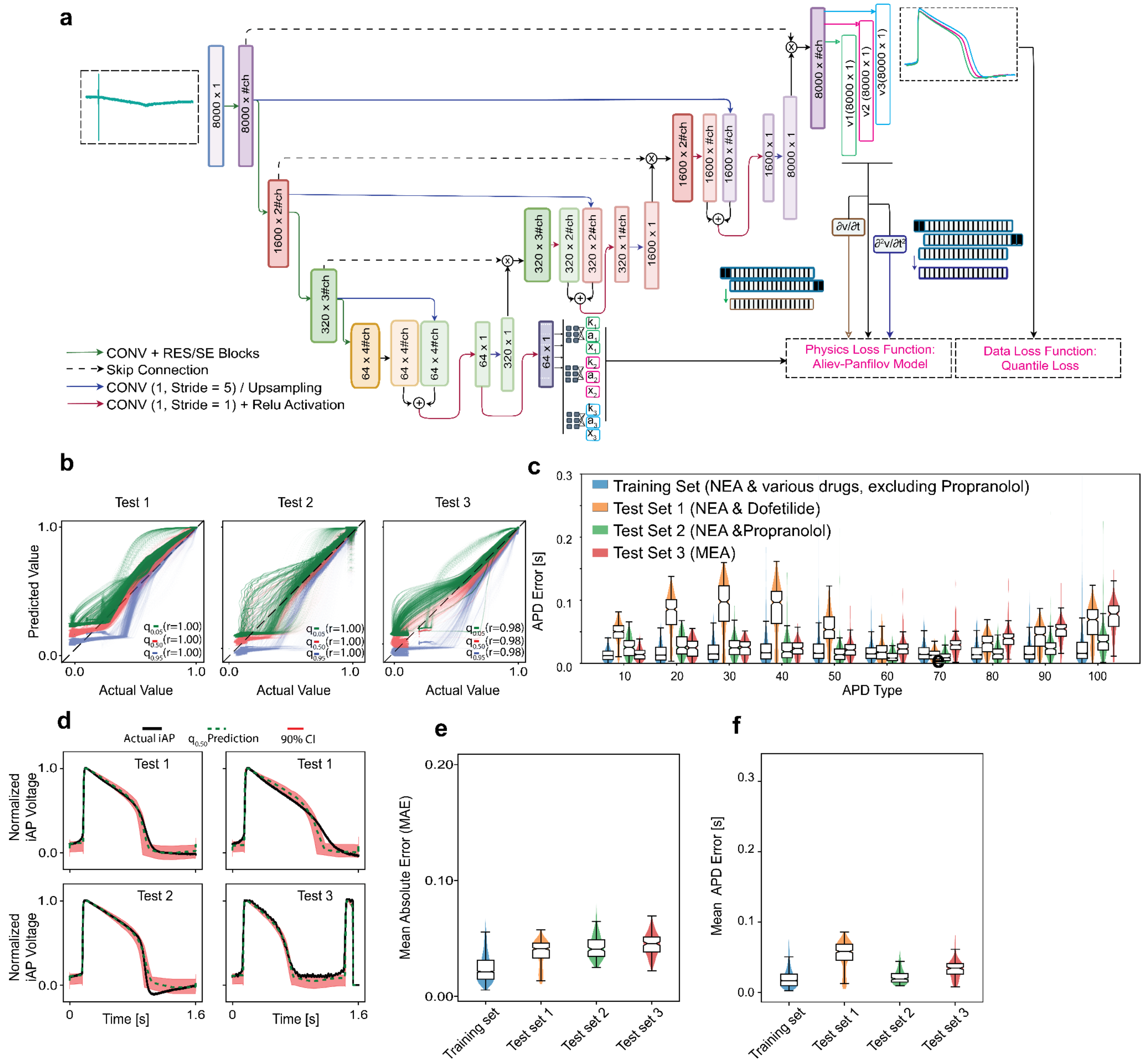

Figure-S12. Qunatille-Physics-Informed Attention Unet (QPIA-UNET) can reconstruct the entire iAP waveform from the eAP signal with confidence interval. a) QPIA-UNET Model Schematic: This provides a visual representation of the QPIA-UNET model used in the study. The model simultaneously predicts three potentials (V_1, V_2, V_3) represented in green (V1, q = 0.05), red (V2, q = 0.5), and blue (V3, q = 0.095), along with their corresponding physics parameters (k_1, x_1 and a_1 in green, k2, x_2 and a2 in red, and k3, x_3 and a3 in blue) from the bottleneck. **b)** This illustrates how the model's predicted potential values closely align with actual values, in three different tests sets, achieving a correlation of r = 1.00, 1.00 and 0.98, respectively on test1, test2 and test3.. **c)** Reconstructed iAPs from the median predictor (q = 0.5) are compared with actual ones in terms of APD. **d)** examples of predicted iAP with their CI (confidence interval) vs the actual ones. **e)** The model exhibits a MAE of 0.04 ± 0.01 on Test 1, 0.04 ± 0.01 on Test 2, and 0.05 ± 0.01 on Test 3. The MAE on the training set is 0.02 ± 0.01, indicating lower error during training. **f)** The mean absolute APD error from **APD10 to APD100** was **0.05 ± 0.04 s** for **Test 1**, **0.02 ± 0.02 s** for **Test 2**, and **0.03 ± 0.03 s** for **Test 3**, with the training set showing a lower error of **0.02 ± 0.02 s**. **In terms of percentage error, these values correspond to 13.07± 13.45% for Test 1, 4.89 ± 5.29% for Test 2, 6.95 ± 4.36% for Test 3, and 4.49 ± 4.99% for the training set.**

References

1. Jahed, Z. *et al.* Nanocrown electrodes for parallel and robust intracellular recording of cardiomyocytes. *Nat. Commun.* **13**, 2253 (2022).
2. Marschang, H., Beyer, T., Karolyi, L., Kübler, W. & Brachmann, J. Differential rate and potassium-dependent effects of the class III agents d-sotalol and dofetilide on guinea pig papillary muscle. *Cardiovasc. Drugs Ther.* **12**, 573–583 (1998).
3. Zeng, A., Chen, M., Zhang, L. & Xu, Q. Are Transformers Effective for Time Series Forecasting? *AAAI* **37**, 11121–11128 (2023).
4. Ching, T., Zhu, X. & Garmire, L. X. Cox-nnet: An artificial neural network method for prognosis prediction of high-throughput omics data. *PLoS Comput. Biol.* **14**, e1006076 (2018).
5. Ainscough, B. J. *et al.* A deep learning approach to automate refinement of somatic variant calling from cancer sequencing data. *Nat. Genet.* **50**, 1735–1743 (2018).
6. Brendel, M. *et al.* Application of Deep Learning on Single-cell RNA Sequencing Data Analysis: A Review. *Genomics Proteomics Bioinformatics* **20**, 814–835 (2022).
7. Yin, Z. *et al.* Neural networks prediction of the protein-ligand binding affinity with circular fingerprints. *Technol. Health Care* **31**, 487–495 (2023).
